# Measurement of shear wave speed dispersion in the placenta by transient elastography: A preliminary *ex vivo* study

**DOI:** 10.1371/journal.pone.0194309

**Published:** 2018-04-05

**Authors:** Emmanuel G. Simon, Samuel Callé, Franck Perrotin, Jean-Pierre Remenieras

**Affiliations:** 1 UMR 1253, iBrain, University of Tours, Inserm, Tours, France; 2 Department of Obstetrics, Gynecology and Fetal Medicine, University Hospital Center of Tours, Tours, France; 3 GREMAN, UMR CNRS 7347, University of Tours, Tours, France; Universite de Nantes, FRANCE

## Abstract

**Background:**

Placental elasticity may be modified in women with placental insufficiency. Shear wave elastography (SWE) can measure this, using acoustic radiation force, but the safety of its use in pregnant women has not yet been demonstrated. Transient elastography (TE) is a safer alternative, but has not yet been applied to the placenta. Moreover, the dispersion of shear wave speed (SWS) as a function of frequency has received relatively little study for placental tissue, although it might improve the accuracy of biomechanical assessment.

**Objective:**

To explore the feasibility and reproducibility of TE for placental analysis, to compare the values of SWS and Young’s modulus (YM) from TE and SWE, and to analyze SWS dispersion as a function of frequency *ex vivo* in normal placentas.

**Materials and methods:**

Ten normal placentas were analyzed *ex vivo* by an Aixplorer ultrasound system as shear waves were generated by a vibrating plate and by using an Aixplorer system.

The frequency analysis provided the value of the exponent n from a fractional rheological model applied to the TE method. We calculated intra- and interobserver agreement for SWS and YM with 95% prediction intervals, created Bland-Altman plots with 95% limits of agreement, and estimated the intraclass correlation coefficient (ICC).

**Main results:**

The mean SWS was 1.80 m/s +/- 0.28 (standard deviation) with the TE method at 50 Hz and 1.82 m/s +/-0.13 with SWE (*P* = 0.912). No differences were observed between the central and peripheral regions of placentas with either TE or SWE. With TE, the intraobserver ICC for SWS was 0.68 (0.50–0.82), and the interobserver ICC for SWS 0.65 (0.37–0.85). The mean parameter n obtained from the fractional rheological model was 1.21 +/- 0.12, with variable values of n for any given SWS.

**Conclusions:**

TE is feasible and reproducible on placentas *ex vivo*. The frequency analysis of SWS provides additional information about placental elasticity and appears to be able to distinguish differences between placental structures.

## Introduction

In clinical practice, placental function is generally assessed by the following standard ultrasound (US) measurements: fetal growth, amniotic fluid index, fetal Doppler indexes (umbilical artery, middle cerebral artery, ductus venosus, and aortic isthmus), and the uterine artery Doppler. The placenta is usually considered primarily from a hemodynamic perspective, but can also be studied as a tissue by investigating its biomechanical properties. US elastography is a relevant tool for such an analysis, because it makes it possible to observe by US waves the deformation of an environment subjected to a constraint. This technology has already been successfully applied in many fields of medicine, such as hepatology [1,2], breast cancer [3], and renal [4], prostate [5] and thyroid conditions [6]. One of the other organs that are candidates for elastography (for example, skin [7], vessels [8], and brain [9]) is the placenta. Moreover, evidence supports the hypothesis that placental insufficiency (preeclampsia or intrauterine growth restriction (IUGR)) may modify elasticity. Significant changes in placental microarchitecture have already been described in these situations, including, for example, increases or decreases in the number of villi ramifications and in fibrin deposits in term villi [10–18]. These changes may affect the stiffness of the entire placenta, probably from early pregnancy, and can potentially be detected by elastography.

**Fig 1:**
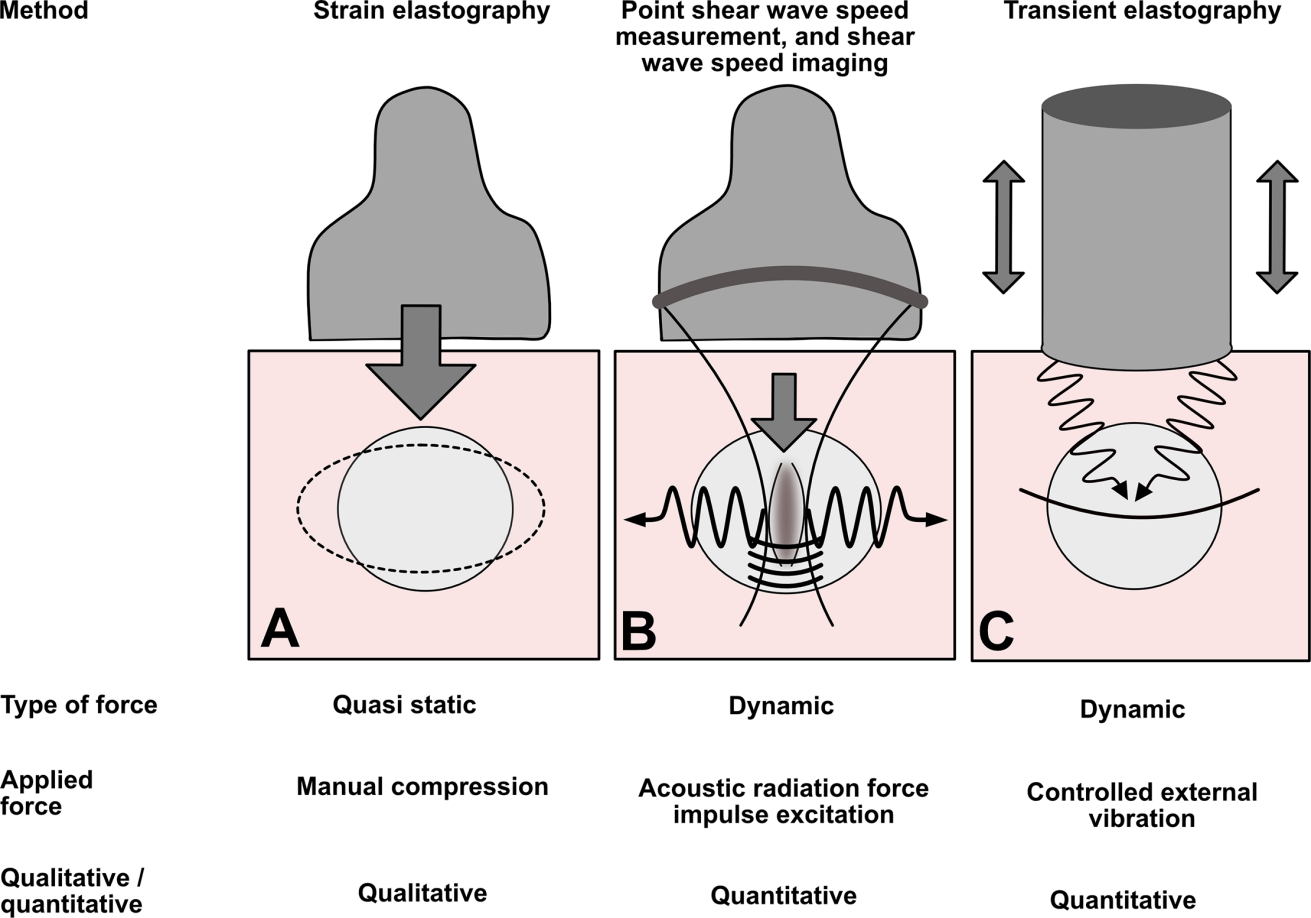
Main elastography methods. In strain elastography (or quasi static elastography) (A), the operator press the tissue with imaging transducer. In the second case, the shear wave is generated by acoustic radiation force impulse excitation. Several methods use this technique: point shear wave speed measurement (average shear wave speed in a region of interest) or shear wave speed imaging (in ShearWave™ Elastography *SWE*™, pushes are applied in several zones, and the corresponding structure of diffraction is controlled) (B). The last method (C) is transient elastography: the shear wave pulse is generated by a surface impulse—thumper. In clinical practice, this technique is used by the Fibroscan™ 1-D system (Echosens, France), especially for the application of liver stiffness measurement.

Some authors have recently studied the placenta by US elastography [19–36], but we propose a new method for this analysis. The field of elastography covers many different US techniques, based on physical approaches that are sometimes quite distant from one another [37–40] (Fig 1). Two basic methods of elastography can be distinguished: quasi static and dynamic. The first, quasi static elastography, measures relative deformation of the target (strain *ε*), most often by applying a stress T with the probe. This method use the linear relation between stress and strain in an isotropic elastic tissue (*T* = *Eε*) with E the Young modulus. The inverse problem $E=\frac{T}{\varepsilon }$ is difficult to solve because the stress T is unknown inside the tissue (T is only known at the surface of the body). The stiffness is then estimated qualitatively by measuring only the strain *ε* everywhere in the region of interest. The second, dynamic elastography, involves generating a shear wave and measuring its speed (shear wave speed: *SWS*) by an ultrafast US device to deduce the elastic modulus (most of the time, ultrafast imaging is necessary). There are two possible ways to generate this shear wave: either radiation force (point SWS measurement, also known as acoustic radiation force impulse *ARFI* quantification, or SWS imaging, for example ShearWave™ Elastography SWE™, Supersonic imagine, France), or external vibrators (shakers/actuators) for transient elastography (*TE*). Recently, ARFI and Supersonic Imaging *SSI* have been used for placental exploration [20–22,27,34,35], but the safety of applying US radiation force *in vivo* to pregnant women has not yet been demonstrated [41]. These methods generate a considerably higher thermal index (TI) than a conventional US examination does. For that reason, some authors have studied SWE™ on animals (pregnant baboons) rather than pregnant women [23,42]. Although the early data appear reassuring, it is currently too early to generalize the use of these techniques on pregnant women and their fetuses. TE methods, on the other hand, use US only in imaging mode and thus have the advantage of generating fewer thermal and mechanical effects than radiation force methods (which use US in both imaging and push modes). For that reason, they may be safer for the fetus. The use of external shakers/actuators for US elastography is common in other areas of medicine (e.g., in hepatology [43]) but not in obstetrics. Nonetheless, similar devices could be developed for pregnant women. Operators could place them on the woman’s abdomen, facing the uterus, while a US probe records the SWS.

TE methods have thus far not been applied to the placenta. While waiting to complete the safety data for the use of this type of vibrating shaker in pregnant women, we propose an *ex vivo* study of delivered placentas to assess the feasibility of a new 2D TE method and compare its results with those of SWE.

The second aim of our study is to analyze the SWS variation according to the frequency. Theoretical and physical considerations indicate that microscopic obstacles in a tissue may influence not only the absolute value of viscoelastic tissue parameters, but also their relation to frequency [44]. To date, among the elastographic methods that have been applied to the placenta, only one of them has analyzed the SWS as a function of frequency: a method called Shear Wave Absolute Vibro-Elastography (SWAVE) [36]. In our study, we propose to use a new method, different from SWAVE, to perform this frequency analysis. We hypothesize that this type of frequency analysis of SWS would provide additional information about the structure of the placental tissue. If so, it may be possible to improve the identification of placental insufficiency by using a tool more suitable for this tissue. In this preliminary feasibility study, we considered only normal placentas.

## Materials and methods

### Study population

The study sample included 10 normal placentas from pregnant women who gave birth vaginally between 37 and 41 weeks’ gestation. The local ethics committee Espace de Réflexion Ethique Région Centre *ERERC* specifically approved this study (RNI 2017–037). Participants provided their verbal informed consent. This consent was collected and archived in the study data by two of the authors (ES and FP). Written consent was not sought in accordance with French legislation. The procedure for obtaining consent was approved by the ethics committee. These women had no diseases, had singleton pregnancies, a normal-weight fetus during previous US scans (estimated fetal weight between the 10^th^ and 90^th^ percentile), a normal amniotic fluid index, and normal fetal Doppler findings (pulsatility indexes of the umbilical artery and the middle cerebral artery). The exclusion criteria were cesarean delivery, manual removal of retained placenta, preexisting or gestational diabetes mellitus, preexisting or gestational hypertension, preeclampsia, sonographic suspicion of IUGR, small-for-gestational-age newborn (birth weight below the 10^th^ percentile), macrosomia (birth weight above the 90^th^ percentile), fetal malformation, uterine malformation, or multiple pregnancy. In all cases, the gestational age was determined from the first trimester US scan, between 11 and 14 weeks’ gestation.

Table 1 presents the clinical characteristics of the study population.

**Table 1:** Clinical characteristics of the study population.

Clinical features	Study population
Maternal age (years) Mean +/- SD	30.7 +/- 4.08
Parity	
- Nulliparous women n (%)	- 5 (50.00)
- Parous women n (%)	- 5 (50.00)
BMI: mean +/-SD	21.99+/- 2.22
Median gestational age at delivery (weeks + days) (range)	39 + 6 (38 + 1; 40 + 2)
Birth weight (g): mean +/- SD	3430 +/- 439
Apgar score (1 min) n (%)	
- 10	- 9 (90.00)
- <10	- 1 (10.00)
- ≤5	- 0 (0.00)
Apgar score (5 min) n (%)	
- 10	- 10 (100.00)
- <10	- 0 (0.00)
- ≤5	- 0 (0.00)
Arterial pH value: mean +/- SD	7.27+/- 0.08
Time of elastographic examination after childbirth (min): mean +/- SD	492 +/- 262

### Multi-frequency elastography system

**Fig 2:**
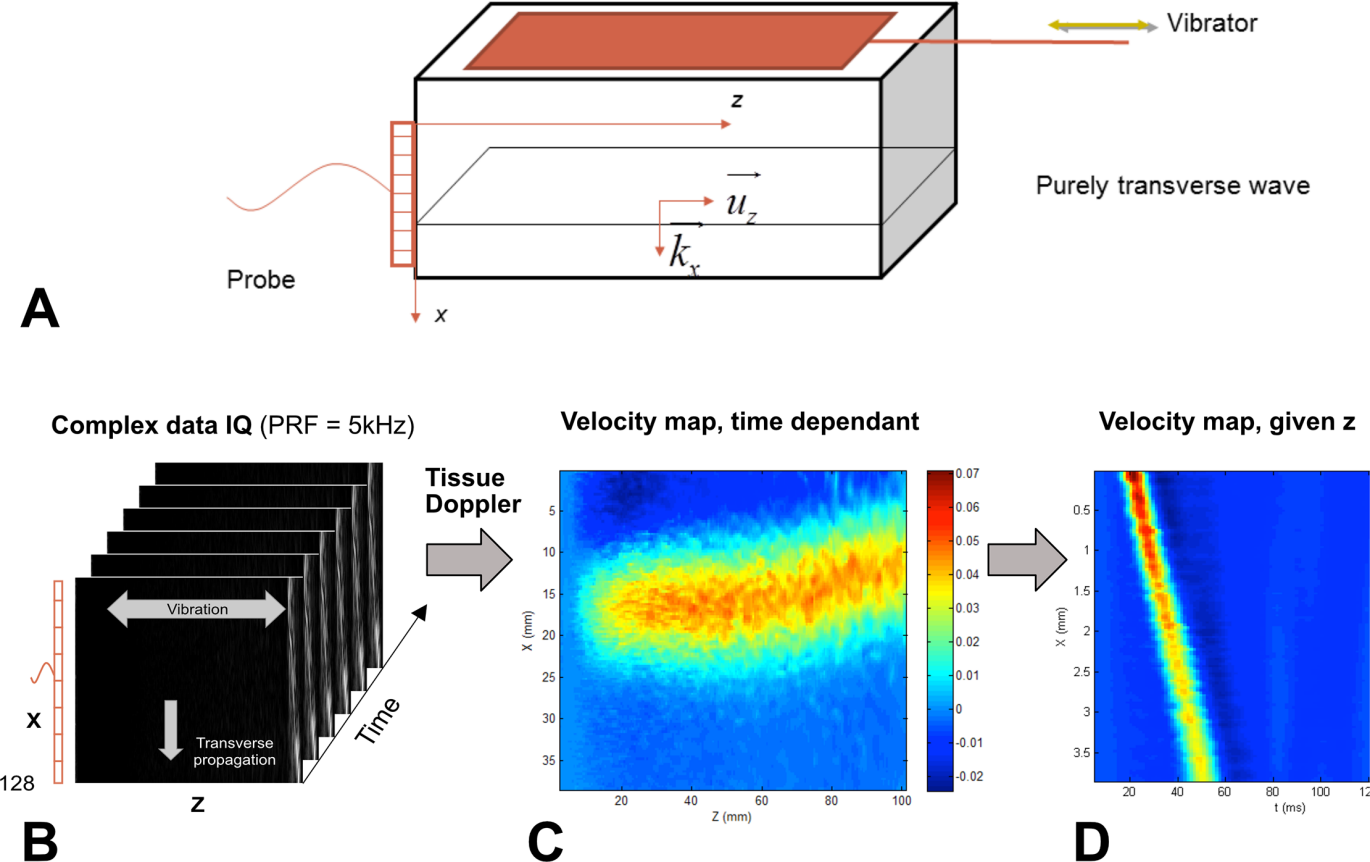
Experiment setup and data processing. The plate moves along the horizontal axis (z axis) (A). The probe is placed perpendicular to it, along the x axis. Beamformed demodulated IQ data is acquired with the scanner (B) and is only sensitive to *V*_*z*_ displacement velocity. Particle velocity map at a given time *t*_0_, confirming the plane nature of the shear wave (C). Particle velocity map for a given *z*_0_(D): *V*_*z*_(*x*,*z*,*t*_0_). The slope of the curve provides a quick approximation of the group velocity. Then, we calculate the spatial FFTs of *V*_*z*_(*x*,*t*) to obtain *V*_*z*_(*x*,*ω*).

**Fig 3:**
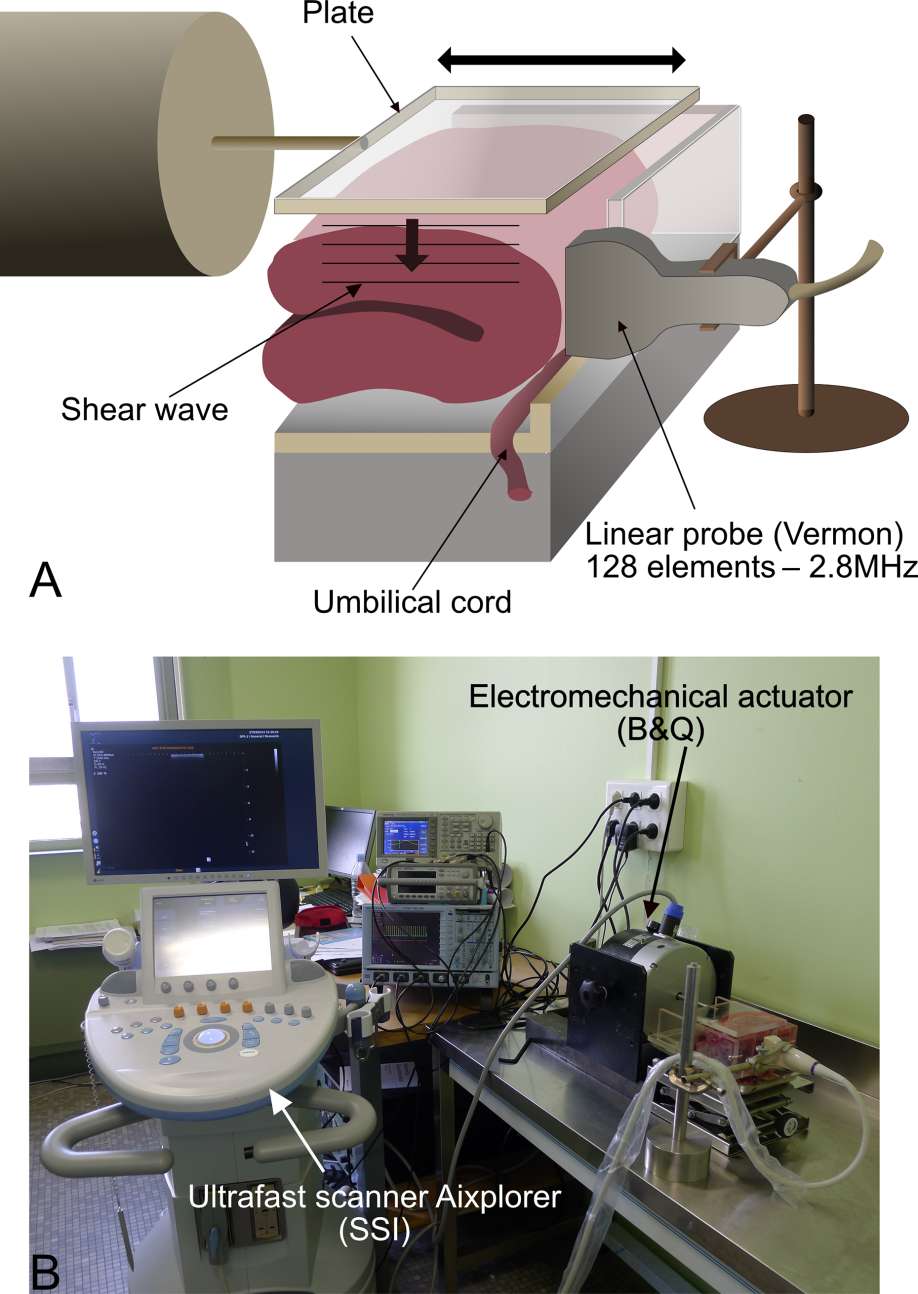
General setup of the experiment. A. A placenta is folded in half in a specially designed box. The umbilical cord is extended out to prevent interference with the measurements. A plate produces the plane shear wave, which is recorded by the Aixplorer™ probe. B. Presentation of the experiment showing the ultrafast US imaging system (Aixplorer™), the US probe, the electromechanical shaker/actuator, and the placenta.

The elastography method we developed is based on a previously published TE method [45], adapted to the placenta and extended to measure the dispersion of the shear modulus. The experimental setup was as follows: a rigid Plexiglas plate was used to produce mechanically a plane shear wave in the placenta (Figs 2 and 3). The plane shear wave propagates in the x direction along the aperture of the US probe. This shear wave is purely transversally polarized, *i*.*e*., the transient displacement induced by the wave to the tissue is only in the z direction. An ultrafast US system (Aixplorer™, Supersonic imagine, France) capable of acquiring 5000 frames per second was used with a 2.8 MHz linear probe (128 elements, Vermon SA, France) to record IQ (in-phase and quadrature) data (Pulse Repetition Frequency: 5 kHz). On each acquisition, the scanner triggered a signal generator (Tektronix AFG 31023), driving an electromechanical actuator (Bruel & Kjaer 4826). The plate was connected to the actuator generating a plane, low amplitude shear wave in the placenta. The signal use to drive the vibrator was a Gaussian shaped wave, providing a single, broadband acquisition [20–80 Hz]. The IQ data are obtained from the Aixplorer™ scanner and we finally recorded a 3D matrix IQ(x,z,t) with $t=n\Delta t_{PRF}=\frac{n}{PRF}$ (Fig 2A and 2B).

The IQ signal can be written:

$$IQ(x,z,t)=I(x,z,t)+jQ(x,z,t)=A(x,z,n)e^{j\phi (x,z,t)}$$

where A is the signal amplitude, *ϕ*(*x*,*z*,*t*) the signal phase at position (x,z) and t the time. An algorithm was developed to compute tissue velocity from the IQ data, based on a subsample Doppler mean frequency estimation method [46]. The velocity estimator is an extension of the autocorrelation estimator developed by Hoeks *et al*. [47] and can be expressed mathematically as follows:

$$V(x,z,t)=\frac{\lambda }{4\pi T}\times arg\left(\sum_{a=0}^{N_{a}}\sum_{b=0}^{N_{b}}IQ(x,z-a,t-b)\bar{IQ}(x,z-a,t-b-1)\right)$$

where *λ* is the US wavelength (550μm) and T the time interval between two acquisitions, $T=\frac{1}{PRF}=200\mu s$. *N*_*a*_ is the number of samples defining the volume of interest (*N*_*a*_ = 30) and *N*_*b*_ the number of temporal samples in the autocorrelation over which the mean velocity is estimated (*N*_*b*_ = 10).

This algorithm estimates the tissue particle velocity V(x,z,t) from the temporal evolution of the IQ data between two successive acquisitions separated by the time $T=\frac{1}{PRF}$. The phase variation is averaged over *N*_*b*_ acquisitions to improve the performances of the algorithm [48]. Hoeks *et al*. proposed to average also the phase estimation over *N*_*a*_ spatially contiguous IQ samples in spatial dimension [47]. In this case the wavelength of the shear wave (*λ*_*S*_ ≃ 2*cm*) have to be much higher than the spatial resolution of IQ data to obtain local resolve estimates. Finally, we obtain a movie of the shear wave propagation (Fig 2C) *V*_*z*_(*x*,*z*,*t*) from this tissue Doppler method. From the Doppler equation, we measure the projection of the tissue particle velocity *V*_*z*_ along the direction of propagation of IU, *i*.*e*. on the z direction. This direction is exactly the direction of the motion induced by the shear wave. We select a specific *z*_0_ position and analyze the propagation of the shear wave following *V*_*z*_(*x*,*t*,*fixed position z*_0_) (Fig 2D). After Fourier transform, we obtain *V*_*z*_(*x*,*ω*) and we can analyze for different fixed pulsations *ω*_*f*_ the tissue particle velocity *V*_*z*_(*ω*_*f*_,*x*) assuming a plane wave model:

$$V_{z}(\omega_{f},x)=V_{0}(\omega_{f})e^{j\frac{\omega_{f}}{c_{s}(\omega_{f})}x}e^{-\alpha (\omega_{f})x}$$

with *f* the frequency, *ω*_*f*_ a fixed pulsation (*ω* = 2*πf*), and *α* the attenuation coefficient.

This device has been further modified and adapted for *in vivo* use (S1 Appendix). This new TE system is based on the propagation of a shear wave generated by two vibrating rods placed on either sides of the US probe (same probe as before). The electrodynamical exciters (Visaton, Germany) are completely decoupled from the US probe and generate the vibration of the two rods. In this configuration, the shear wave is generated in the vibration direction in front of the US probe elements, between the two rods. The shear wave propagation, measured by the US probe, is due to the constructive interference between the two waves generated by the two rods.

### Microscopic architecture, shear wave dispersion, and rheology

**Fig 4:**
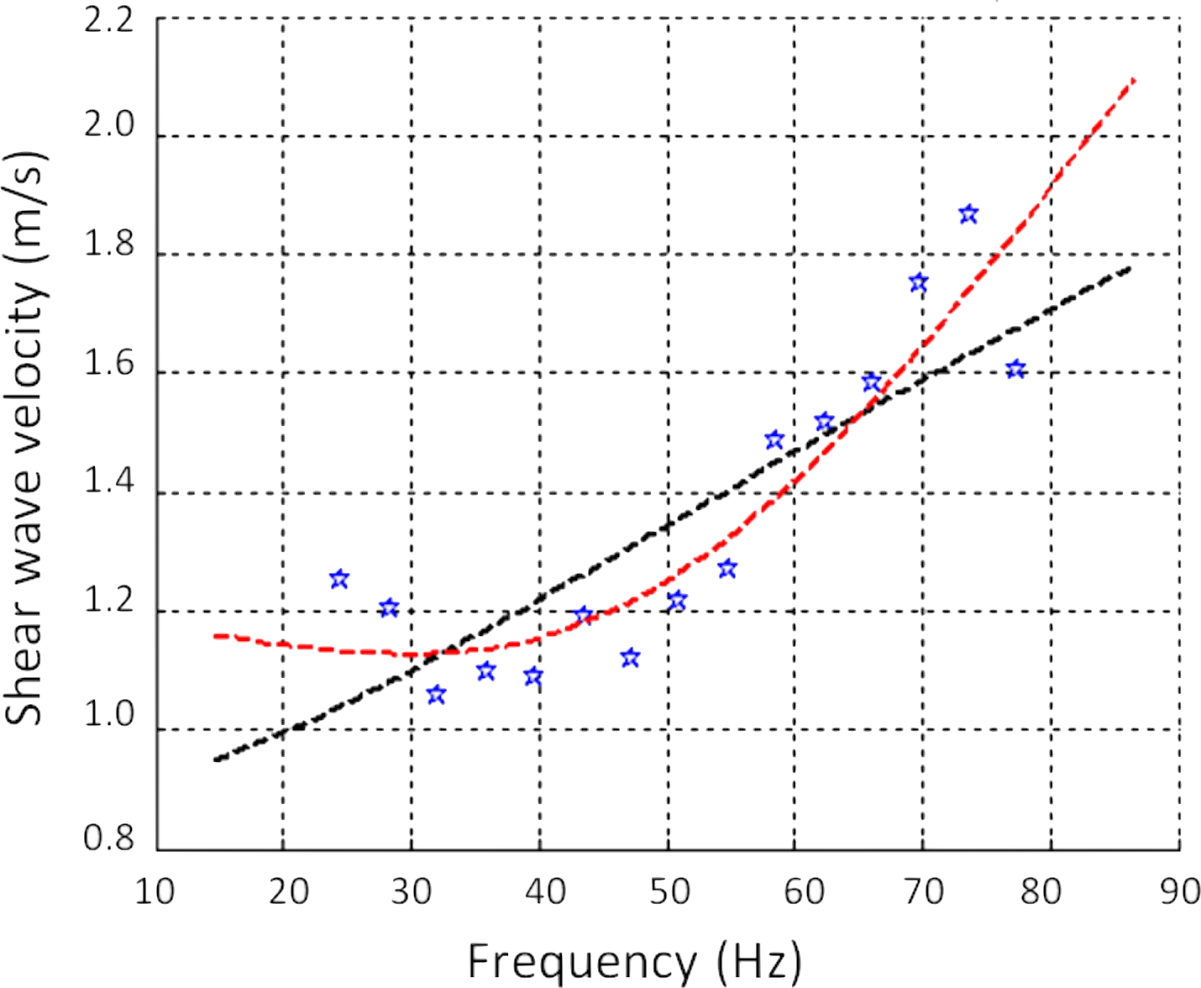
Dispersion of the shear wave speed according to frequency in one placenta. Blue points are the experimental data. The black dashed line represents the Voigt model, which is widely used in elastography, but does not accurately simulate viscoelastic behavior as a function of frequency variation. The red curve is a fractional rheological model, which fits the data better; n, its exponent, provides a simple quantitative interpretation of the measurements.

Theoretical and physical considerations suggest that the presence of microscopic obstacles influences not only the absolute value of viscoelastic tissue parameters, but also their relation to frequency. The propagation of shear waves in a viscoelastic medium is physically associated with the viscoelastic properties of the medium. In particular, the frequency behavior of tissue mechanical parameters can be modeled as a power law; it cannot, however, be modeled by the classical Voigt or Maxwell viscoelastic models widely used in elastography (Fig 4). We used a fractional rheological model that is capable of describing power law behavior for the complex shear modulus G*(ω) [49]:

$$G*\left(i\omega \right)=Ge+K.{\left[i\omega \right]}^{n}$$

In this model, G* is the elastic modulus (Pa), Ge is the shear modulus at equilibrium (Pa), K is a constant (consistency coefficient, Pa.s^n^), and n is a linear parameter (without unit). This exponent parameter n represents a mechanical property inherent to a given materiel [44] and was assumed in our study to provide information about the microscopic form of the tissue. Measurements were made in the bandwidth [20 Hz-80 Hz].

This method has been validated on calibrated viscoelastic gels [46]

### Description of the protocol

The placentas were analyzed < 12 hours after delivery (mean time: 8 h, Table 1). The placental membranes were resected, and the umbilical cord was extended out, horizontally, so that only the shear wave propagation within the cotyledons would be analyzed. The placenta was then placed in a specially designed box (Fig 3). Detection of the plane shear wave required that the probe be positioned perpendicularly to the fetal surface of the placenta. Because the placenta was too thin for this, however, it was folded over, in half. If we had not folded it, our area of exploration would have been very limited, and reflections of the support could have constituted an additional difficulty.

Measurements for each placenta were made in two different regions: in the central region (near the umbilical cord) and in the peripheral region (on the placental border). Accordingly, the placental position in the box was modified after the first measurement.

IQ data were recorded for each measurement, and the complex shear modulus was determined. In addition, Young's modulus (YM) and SWS were calculated with the SSI method for comparison with the values of our TE method.

In the SSI method, the central frequency of the shear wave depends on the duration of the push. The characteristics of this pulse are very variable, for example with the explored organ, and constitute information internal to the system. We did not have access to this frequency information. To compare the SSI and TE methods, we considered the SWS values at 50 Hz in TE, as it was the central frequency of the excitation signal of the shaker/actuator.

Each measurement (from TE and SSI) was repeated 3 times by repositioning the probe each time, and with two different operators (ES and SC). It was thus possible to calculate intra- and inter-operator reproducibility.

### Statistical analysis

Categorical data are presented as numbers and percentages (n (%)), and continuous data as means with their standard deviations. All comparisons used the Mann–Whitney U-test. To assess intra- and inter-observer reproducibility, we calculated intraclass correlation coefficients (ICC). Bland-Altman plots with 95% intervals of agreement were generated for SWS and YM.

Differences were considered significant for *P*-values <0.05. R 3.3.2 software was used to perform the analyses.

## Results

Measured with the SSI method, the mean YM value was 10.50 kPa +/- 1.73 in the central regions and 10.60 kPa +/- 2.17 in the peripheral regions (*P* = 0.791) (Table 2 and S1 Table). Measured with our new TE method, the corresponding YM values were 11.34 kPa +/- 4.49 in the central and 9.78 kPa +/-2.72 in the peripheral location (*P* = 0.579, and not significant for comparison between SSI and TE, *P* = 0.796 for the central and 0.384 for the peripheral regions).

**Table 2:** Values of Young’s modulus, shear wave speed at 50 Hz, and n among 10 normal placentas, *ex vivo*.

	Method and comparison	General	Central region	Peripheral region	Comparison central/peripheral: *P* value
E (kPa) Mean+/-standard deviation	SSI	10.57 +/- 1.53	10.50 +/- 1.73	10.60 +/- 2.17	0.791
	TE	10.56 +/- 3.25	11.34 +/- 4.49	9.78 +/- 2.72	0.579
	*P* value	0.912	0.796	0.384	
SWS (m/s) 50 Hz Mean+/-standard deviation	SSI	1.82 +/- 0.13	1.82 +/- 0.15	1.82 +/- 0.18	0.684
	TE	1.80 +/- 0.28	1.86 +/- 0.39	1.75 +/- 0.24	0.570
	*P* value	0.912	0.791	0.436	
n Mean+/-standard deviation	TE	1.21 +/- 0.12	1.15 +/- 0.18	1.25 +/- 0.11	0.069

E (Young’s modulus), SSI (supersonic shear imaging), SWS (shear wave speed), TE (transient elastography), n (exponent of the fractional rheological model)

All comparisons were tested with the Mann–Whitney *U*-test

The mean SWS values from SSI were 1.82 m/s +/- 0.15 (central) and 1.82 m/s +/- 0.18 (peripheral) (*P* = 0.684), and the corresponding values from TE were 1.86 m/s +/- 0.39 (central) and 1.75 m/s +/- 0.24 (peripheral) (*P* = 0.570). The SSI/TE comparisons were not significant either: *P* = 0.791 and 0.436 for the central and peripheral regions. The mean value for the n parameter calculated from TE was 1.15 +/- 0.18 in the central and 1.25 +/- 0.11 in the peripheral regions (*P* = 0.069).

Table 3 and S1 Table present the SWS values calculated by both methods (SSI/TE) and the values of n (TE) for each placenta.

**Table 3:** Values of the shear wave speed from supersonic shear imaging and transient elastography.

	Central region			Peripheral region		
Placenta	SWS SSI (m/s) +/- *SD*	SWS TE (m/s)+/- SD	n+/- SD	SWS SSI (m/s) +/- *SD*	SWS TE (m/s) +/- *SD*	n +/- *SD*
1	1.82+/- *0*.*10*	2.27+/-*0*.*47*	1.17+/-*0*.*06*	1.76+/-*0*.*09*	1.87+/-*0*.*19*	1.10+/-*0*.*05*
2	1.86+/-*0*.*16*	2.26+/-*0*.*05*	1.09+/-*0*.*07*	1.83+/-*0*.*23*	2.21+/-*0*.*04*	1.25+/-*0*.*02*
3	1.63+/-*0*.*02*	2.10+/-*0*.*08*	1.06+/-*0*.*08*	1.60+/-*0*.*30*	2.00+/-*0*.*12*	1.18+/-*0*.*05*
4	1.79+/-*0*.*18*	1.76+/-*0*.*27*	1.08+/-*0*.*02*	1.89+/-*0*.*09*	1.49+/-*0*.*04*	1.36+/-*0*.*04*
5	2.04+/-*0*.*30*	1.32+/-*0*.*19*	1.15+/-*0*.*04*	1.70+/-*0*.*09*	1.60+/-*0*.*37*	1.38+/-*0*.*14*
6	1.58+/-*0*.*41*	2.17+/-*0*.*21*	1.25+/-*0*.*06*	1.90+/-*0*.*48*	1.80+/-*0*.*04*	1.32+/-*0*.*10*
7	1.99+/-*0*.*35*	1.60+/-*0*.*11*	1.52+/-*0*.*08*	1.83+/-*0*.*37*	1.55+/-*0*.*05*	1.28+/-*0*.*13*
8	1.90+/-*0*.*09*	1.49+/-*0*.*07*	1.31+/-*0*.*03*	1.70+/-*0*.*16*	1.86+/-*0*.*24*	1.35+/-*0*.*07*
9	1.72+/-*0*.*19*	1.40+/-*0*.*13*	0.96+/-*0*.*39*	1.86+/-*0*.*22*	1.46+/-*0*.*05*	1.08+/-*0*.*22*
10	1.98+/-*0*.*24*	2.27+/-*0*.*25*	0.93+/-*0*.*19*	2.26+/-*0*.*03*	1.66+/-*0*.*18*	1.25+/-*0*.*09*

SSI (Supersonic Shear Imaging), SWS (Shear Wave Speed), TE (Transient Elastography)

**Fig 5:**
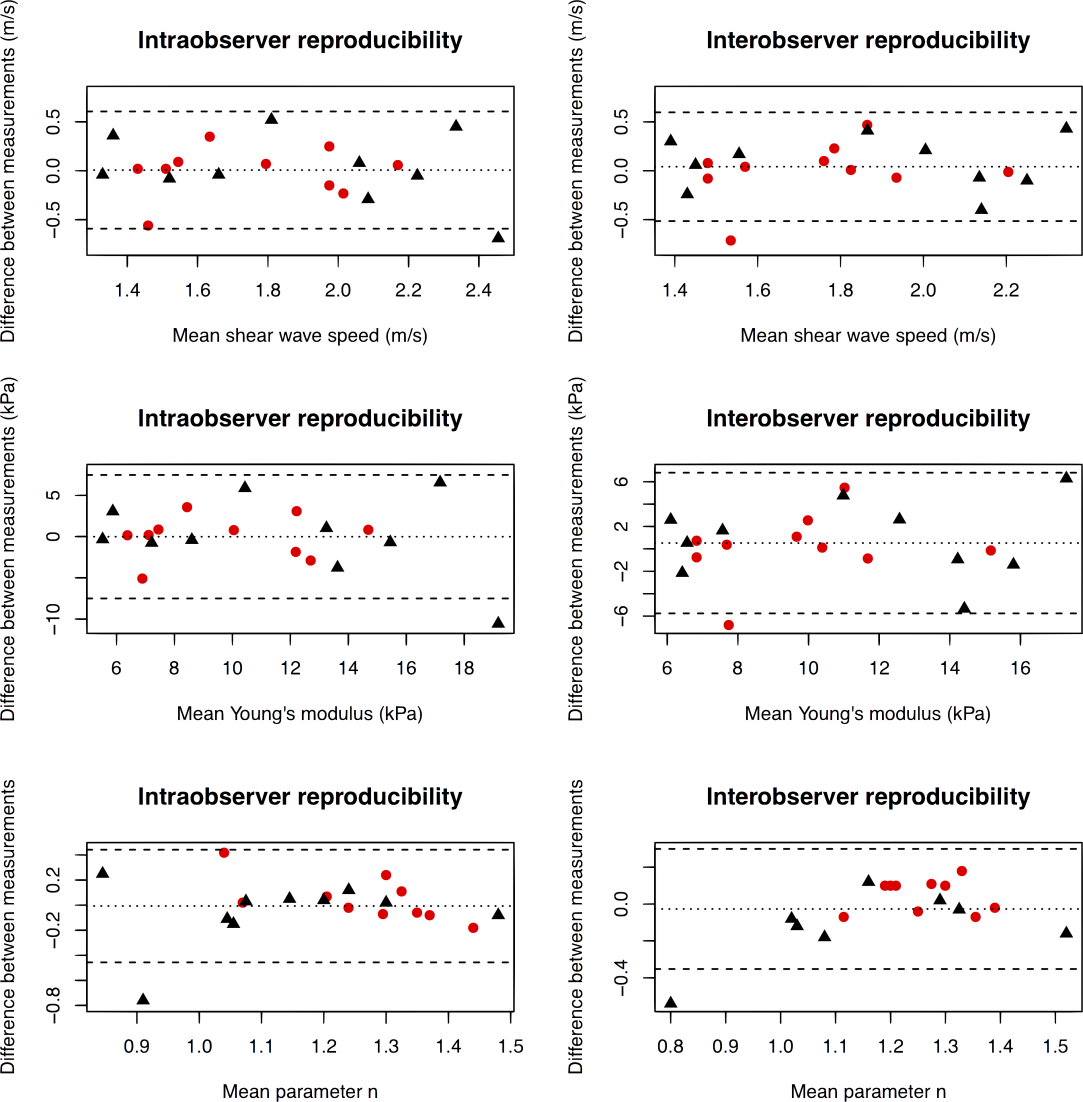
Intraobserver and interobserver variability of Young’s modulus, shear wave speed, and parameter n values with 95% limits of agreement. Measurements in the central region of the placenta are represented by black triangles (▲) and those in the peripheral region by red dots (●).

For TE, reproducibility was good for the calculation of SWS, both for the same and different examiners (ICC = 0.68 (95% CI 0.50, 0.82), and 0.65 (95% CI 0.37, 0.85) respectively)(Table 4). This reproducibility of SWS was lower for SSI (0.54 (95% CI 0.09, 0.75) and -0.13 (95% CI -0.59, -0.35) respectively). Bland-Altman plots are shown in Fig 5.

**Table 4:** Reproducibility of Young’s modulus and shear wave speed from supersonic shear imaging and transient elastography.

Method: SSI or TE	E or SWS	Intraobserver variability		Interobserver variability	
		95% limits	ICC (95% CI)	95% limits	ICC (95% CI)
SSI	E (kPa)	-5.37, -5.45	0.54 (0.07, 0.76)	-9.80, -7.16	-0.09 (-0.57, -0.42)
	SWS (m/s)	-0.48, -0.49	0.54 (0.09, 0.75)	-0.84, -0.62	-0.13 (-0.59, -0.35)
TE	E (kPa)	-7.51, -4.47	0.64 (0.48, -0.81)	-5.76, -6.81	0.66 (0.41, 0.82)
	SWS (m/s)	-0.59, -0.61	0.68 (0.50, 0.82)	-0.51, -0.60	0.65 (0.37, 0.85)
	Parameter n	-0.46, -0.44	0.39 (0.02, 0.80)	-0.35, -0.30	0.60 (0.42, -0.80)

E (Young’s modulus), SSI (supersonic shear imaging), SWS (shear wave speed), TE (transient elastography)

YM and SWS values measured at a fixed frequency by SWE and TE were of the same order of magnitude for both regions (Table 2). Moreover, the value of the parameter n appeared to be independent of the YM and SWS values. In the peripheral regions of placentas n°1 and n°8 (Table 3), the SWS values calculated from TE were very similar: 1.87 m/s +/- 0.19 (placenta n°1) and 1.86 m/s +/- 0.24 (placenta n°8), but the corresponding parameters n obtained from the fractional rheological model were quite different: 1.10 +/- 0.05 (placenta n°1) and 1.35 +/- 0.07 (placenta n°8). The SWS (TE method) in the central regions of placentas n°1 and n°10, were also similar: 2.27 m/s +/- 0.47 (placenta n°1) and 2.27 m/s +/- 0.25 (placenta n°10), while their n values were 1.17 +/- 0.06 and 0.93 +/- 0.19 respectively. In these examples, a difference in the SWS value of a few hundredths corresponded to a difference of a few tenths for n. This difference in these orders of magnitude suggests that the parameter n constituted additional information about placental elasticity.

## Discussion

### Interpretation of main findings

Our study is the first application of a 2D TE method to the placenta. This method is feasible and reproducible on delivered normal placentas. The elastic parameters calculated at 50 Hz were quite similar to those obtained by SWE. The small differences observed can be explained by the fact that TE values were calculated at 50 Hz, which probably did not correspond to the same frequency in SWE. Moreover our method provides information about the variation law of these parameters as a function of frequency. This information is given by the exponent parameter of the power law, called “parameter n”. Values of n should be interpreted as a result independent of the SWS and the YM, but specific to a given tissue architecture. This quantitative value of n can be simply interpreted for solids: the more n tends to 0, the more elastic its behavior, and conversely, the more n deviates from 0, the more viscous its behavior.

Recently, Abeysekera *et al*. published a multifrequency analysis of SWS in placenta using a 3-D motorized probe connected to an Ultrasonics SonixTouch platform with a conventional B-mode ultrasound transmission sequence [36]. The SWAVE system applies a longitudinal vibration to the surface of the placenta using a 3cm diameter circular steel plate connected to a voice exciter. This system uses monochromatic harmonic excitation to generate shear waves like in Magnetic Resonance Elastography and five excitation frequencies at 60, 80, 90, 100 and 120Hz are sequentially emitted. Since the radiated shear wave is not spread in a large bandwidth like in TE, this method allows a good penetration of the shear wave in the placenta even at 120Hz where we are limited at 80Hz in our experiments. The disadvantage is to repeat several times the measurement for each frequency unlike our TE method where the duration of the measurement is 51ms.

Regardless of the method used, TE or SWE, the orders of magnitude of SWS and YM in our study were consistent with previously published data (Table 5). In addition, consistent with the literature, we found no significant difference between the central and peripheral regions of these placentas [20–22,29].

**Table 5:** Literature review of placental elastography.

Reference	Method	Placentas	Results	Histopathological evaluation	Safety data
Animal studies					
Quarello E. *et al*, 2015 [23]	SWE	21 pregnant baboons. Second half of pregnancy.	Reproducibility study. Intra- and inter-observer ICC: (i) for single values, respectively 0.657 (95% CI 0.548 to 0.752) and 0.458 (95% CI 0.167 to 0.675); (ii) for mean values: 0.852 (95% CI 0.784 to 0.901) and 0.628 (95% CI 0.286 to 0.806).	Not reported	The offspring of these pregnancies were born without incident and the clinical follow-up was normal (10 months).
Quibel T. *et al*, 2015 [24]	SWE	18 Sprague Dawley rats. 217 feto-placental units. Ligation of the left uterine artery on embryonic day 17, ultrasound and elastography on embryonic day 19.	Mean YM+/-SD: Left horn 11.7 kPa +/- 1.5; Right horn 8.01 kPa +/- 3.8 (*P <* 0.001). Relation between placental elastography and fetal weight (*r* = 0.42; *P <* 0.001).	Yes	High rate of fetal mortality due to surgical ligation
*Ex vivo* studies					
Sugitani M. *et al*, 2013 [20]	ARFI	115 placentas (26–41 weeks gestation): 74 normal, 24 IUGR, 17 PIH.	Mean SWS values +/- SD. Normal: 1.31 m/s +/-0.35, IUGR: 1.94 m/s +/-0.74, PIH: 1.49 m/s +/-0.52.	Yes	No apparent histological damage to placental tissue
McAleavey S.A. *et al*, 2016 [25]	SWE	11 women: Uncomplicated term pregnancies. After cesarean deliveries, the placentas were placed in a plastic container with an open perfusion system (as a living placenta). Vasoactive substances employed.	SWS color images heterogeneous. Mean SWS: 1.92 m/s +/- standard error 0.05. After vasoconstrictor or vasodilator, heterogeneous and localized response.	No	Not reported
Durhan G. *et al*, 2017 [26]	SE	55 women:25 with IUGR (median gestational age: 38 weeks 2 days), 30 controls (median gestational age: 39 weeks 2 days).	Elasticity index (EI) and histopathological findings were compared between groups. Greater placental stiffness and more histopathological changes were observed in the IUGR group than in controls (*P*<0.05). Histopathological findings were associated with lower EI values, but no specific pattern of histologic abnormalities was identified except for villitis and delayed villous maturity.	Yes	Not applicable
Abeysekera J.M. et al, 2017 [36]	SWAVE	61 women. 37–41 weeks gestation. Clinically normal placentas. The elasticity and viscosity were estimated through rheological modeling.	SWS (± SD) at: 60 Hz: 1.23 m/s ± 0.44; 80 Hz: 1.67 m/s ± 0.76; 90 Hz: 1.74 m/s ± 0.72; 120 Hz: 1.80 m/s ± 0.78. No difference between placentas with or without abnormalities.	Yes. Microscopic examination: 16 placentas had significant abnormalities.	Not reported
*In vivo* studies					
Cimsit C. *et al*, 2014 [19]	SE	144 women (20–23 weeks’ gestation): 101 normal, 28 with preeclampsia, 15 with mild proteinuria or previous preeclampsia. Posterior-lying placentas excluded.	Elasticity ratio (mean, 95% CI): Normal: 0.9 (0.82–0.97); Preeclampsia: 1.56 (1.12–2.16); Proteinuria or previous preeclampsia: 0.72 (0.58–0.9). *P* <0.0001.	No	Not applicable
Cimsit C. *et al*, 2015 [22]	SWE	129 women (20–23 weeks’ gestation): 101 normal, 28 with preeclampsia. Posterior-lying placentas excluded	Mean elastic modulus (range): Normal: 2.53 kPa (2.29–2.80); Preeclampsia: 7.01 kPa (3.79–13.3). No difference between the center or edge of the placenta.	No	Not reported
Li W.J. *et al*, 2012 [21]	SWE	30 women. Normal pregnancies (28–41 weeks’ gestation). Posterior-lying placentas excluded	Mean elastic modulus +/- SD: Placental edge (15 measurements per case, 30 cases): 7.60 kPa +/- 1.71; Central placenta: (15 measurements per case, 30 cases): 7.84 kPa +/- 1.68; Average for all measurements: 7.70 kPa +/- 1.61. No significant difference between the central placenta and the edge. No correlation with uterine or umbilical PI values.	Not reported	Not reported
Ohmaru T. *et al*, 2015 [34]	ARFI	199 women, 5 groups: 143 normal, 21 with IUGR, 15 with PIH, 13 with collagen disease	The correlation between SWS and gestational weeks was not significant. Mean SWS +/- SD: Normal group: 0.98 m/s +/- 0.21; IUGR: 1.28 m/s +/- 0.39; PIH: 1.60 m/s +/-0.45. SWS was significantly higher for IUGR and PIH. SWS and the expression ratio of collagen fibers were significantly correlated.	Yes	Not reported
Kilic F. *et al*, 2015 [27]	SWE	50 women (second or third trimester): 23 with preeclampsia, 27 normal.	Median elastic modulus (range): Preeclampsia: 21 kPa (3–71); Normal: 4 kPa (1.5–14). *P <* 0.001. Cut-off value maximizing the accuracy of diagnosis: 7.35 kPa. AUC: 0.895 (95% CI 0.791–0.998).	No	Not reported
Alan B. *et al*, 2016 [28]	ARFI	74 women (18–28 weeks): 40 structural anomalies (thickened nuchal translucency, short femur, short humerus, pyelectasis, hyperechoic bowel, echogenic intracardiac focus, choroid plexus cyst, heart defects, omphalocele, ventriculomegaly, and limb abnormalities) or non-structural findings, 34 normal.	Mean SWS +/- SD: Study group: 1.89 m/s +/- 0.7; Control group: 1.59 m/ +/- 0.5. *P* = 0.04.	No	Not reported
Yuksel M.A. *et al*, 2016 [29]	SWE	76 women (mean gestational age at SWE: 30.5 weeks’ gestation): 33 with gestational diabetes mellitus (GDM), 43 healthy pregnant women.	Mean YM+/-SD. Mean elasticity values of both the central and peripheral parts of the placentas were significantly higher in women with GDM (*P*<0.001): Central part: 10.63 kPa +/- 5.97 for women with GDM vs 5.47 kPa +/- 1.74 for control cases. Peripheral part: 10.67 kPa +/- 7.41 for GDM vs 5.23 kPa +/- 1.31 for control cases. The mean elasticity values did not differ between the central and peripheral placentas in either group (*P*>0.05).	No	Not reported
Albayrak E. *et al*, 2016 [30]	SE	70 women (second trimester). This study investigated the ability of the placental strain ratio to predict spontaneous preterm birth (sPTB). Mean gestational age at the time of the procedure: 22.51 weeks’ gestation. sPTB group: 10 women, term birth group: 60 women.	Calculation of muscle-to-placenta strain ratio (MPSR) and fat-to-placenta strain ratio (FPSR). Gestational age at birth was slightly negatively correlated with MPSR (r = -0.300, *P* = 0.012) and moderately negatively correlated with FPSR (r = -0.513, *P*<0.001). The multivariate linear regression analysis showed that the FPSR (β = 0.609, *P* = 0.002) was a significant predictor of sPTB.	No	Not applicable
Wu S. *et al*, 2016 [31]	ARFI	50 healthy pregnant women during the second trimester. 50 healthy pregnant women during the third trimester.	Mean SWS +/- SD: 0.983 m/s +/-0.260. Minimum SWS: 0.63 m/s. Maximum SWS: 1.84 m/s. No significant difference in SWS between the second and third trimesters (0.978 m/s +/-0.255 vs 0.987 m/s +/-0.266, *P* = 0.711).	No	Not reported
Alan B. *et al*, 2016 [32]	ARFI	86 women: 42 with preeclampsia, 44 controls.	Mean SWS (IQR): Preeclampsia group: 1.39 m/s (1.32–1.53); Control group: 1.07 m/s (1.00–1.14). *P* < 0.001. Mean SWS (IQR) among women with preeclampsia: Mild preeclampsia (n = 26): 1.34 m/s (1.31–1.39); Severe preeclampsia (n = 16): 1.56 m/s (1.53–1.59).	No	Not reported
Karaman E. *et al*, 2016 [33]	ARFI	107 women: 38 healthy control subjects, 34 with gestational hypertension, 35 with preeclampsia.	Mean SWS (+/-SD): Controls: 0.91 m/s +/- 0.20; Gestational hypertension:1.27 m/s +/- 0.36; Preeclampsia: 1.93 m/s +/- 0.62. *P* = 0.001. SWS was higher in the preeclampsia group than in the other groups in all three regions of the placenta (fetal edge, maternal edge, central part of the placenta). *P* = 0.01.	No	No new data
Arioz Habibi H. *et al*, 2017 [35]	SWE	84 women: 42 IUGR, 42 controls.	Median YM (IQR): (i) IUGR central part: Maternal side: 28 kPa (16.8–35), Fetal side: 21.5 kPa (13.5–28.3); (ii) IUGR peripheral part: Maternal side: 22 kPa (13.8–31.3), Fetal side: 22.5 kPa (14.8–29.5); (iii) Control group central part: Maternal side: 6 kPa (4.38–7.45), Fetal side: 5 kPa (3.73–6.55); (iv) Control group peripheral part: Maternal side: 5.35kPa (4.78–6.28), Fetal side: 5.3 kPa (4–6.85).	No	Not reported

ARFI: acoustic radiation force impulse, GDM: gestational diabetes mellitus, IUGR: intrauterine growth restriction, AUC: area under the curve, PIH: pregnancy-induced hypertension, IQR: interquartile range, SD: standard deviation, SE: strain Elastography, sPTB: spontaneous preterm birth, SWE: shear wave elastography, SWS: shear wave speed.

### Clinical meaning

The use of placental elastography is based on the hypothesis that placental insufficiency modifies the elasticity of this organ. This is a credible hypothesis already suggested by studies (Table 5). Among this literature, several *ex vivo* studies [20,26,36] and *in vivo* studies [19,22,27,32–35] compared normal and insufficient placentas in humans (either preeclampsia, IUGR or both).

Almost all of these studies found that YM or SWS was elevated in cases of placental insufficiency.

Most of the *in vivo* studies have investigated preeclampsia rather than IUGR (except Ohmaru *et al* [34] and Arioz Habibi *et al* [35]), whereas the *ex vivo* studies have tended to examine IUGR instead. On the whole, although these studies are fairly numerous, their sample sizes have been small and the levels of evidence generally very low.

Moreover, they have employed a diverse range of elastography techniques: strain elastography [19,26,30], ARFI [20,28,32–34], or SWE [21,22,25,27,29]. The orders of magnitude of the measurements sometimes differed from one study to another. For example, the mean YM in preeclampsia cases ranged from 7.01 kPa (range 3.79–13.3) [22] to 21 kPa (range 3–71) [27], and the mean SWS value ranged from 1.34 m/s (IQR 1.31–1.39) [32] to 1.93 m/s (+/- SD 0.62) [33]. In normal placentas *in vivo*, mean YMs have varied from 2.53 kPa (range 2.29–2.80) [22] to 7.84 kPa (+/- SD 1.68) [21] and mean SWS from 0.91 m/s (+/- SD 0.20) [33] to 1.59 m/s (+/- SD 0.5) [28]. The values of SWS or YM found in our study are therefore slightly higher than those found in the literature, even in comparison with other *ex vivo* studies [20,25,26,36]. These heterogeneous results underline the need to improve tools for quantifying placental elasticity.

We can make some pathophysiological assumptions about changes in placental elasticity. The main interest of elastography does not lie in identifying localized placental lesions related to preeclampsia or IUGR. Conventional US can identify or at least call attention to suspicious lesions such as infarcts or hematomas. Furthermore, their absence does not exclude a diagnosis of placental insufficiency (whether preeclampsia or IUGR), and their presence is not pathognomonic of it [50,51]. These lesions are sometimes unrecognized when a large part of the placenta is hidden by the fetus, as in posterior locations. The identification of lesions that affect the entire placenta is a more ambitious challenge, especially if these lesions are not visible on a standard US scan. Among the many abnormalities of the placental parenchyma that have been described, we do not know which ones might give rise to elasticity changes. However, the fractional rheological model that we used is known to be sensitive to a shape factor of the considered tissue [44]. We make the hypothesis that structural modifications of the placental parenchyma, whatever their type, have an impact on SWS dispersion. For example, trophoblastic fibrin deposits, calcifications, and changes in villi phenotype might be related to placental elasticity. Deposition of fibrinoid materials around the syncytium is often observed in case of preeclampsia or IUGR [17]. Furthermore, although an increased risk of adverse maternal and fetal outcomes in cases of preterm placental calcifications has not yet been proven, the studies suggesting such an association cannot be ignored [52]. According to the fetal hypoxia paradigm, fetal capillary morphology may differ in cases of preeclampsia and of IUGR, with and without severe Doppler abnormalities [11]. These changes in cotyledon microarchitecture are not currently accessible by conventional US exploration, but they could be interesting candidates for SWS dispersion analysis. The exponent parameter n of the power law has already been studied in several types of tissue, and different approaches have been used to model the dispersion of the shear modulus. Interesting results have been obtained in breast tumors [53] and liver tissue [54,55], with, for example, a multiparameter approach enabling the characterization of liver fibrosis and inflammation [54]. These results have served as a starting point for studying shear modulus dispersion in the placenta.

### Strengths and limitations

The main strength of our study is to demonstrate the feasibility of placental exploration by TE and thus to pave the way of a new approach for biomechanical investigations of the placenta.

The weakness of this approach is the limited depth of exploration possible with TE—about 8 cm with our probe, although we note that other elastographic techniques are also subject to this depth constraint, and all the studies using SE or SWE have excluded posterior-lying placentas. Nonetheless, the literature suggests that there is no difference in elasticity between the central and peripheral regions of the placenta. In clinical situation in pregnant women, the posterior-lying placenta (46% of the cases in the Torricelli *et al*. cohort [56]) is more difficult to access, and anterior placenta seems to be a better candidate for the TE system that we have developed (S1 Appendix). But under certain conditions, it could be sometimes possible to access an edge of the posterior-lying placenta when the US probe is positioned very laterally on the abdomen (on the woman's lumbar or iliac region). For that reason, the question of placental heterogeneity remains important, and we did not find any differences between central and peripheral regions. In addition, *ex vivo* results should be interpreted with caution, as little is currently known about SWS variations as a function of placental vascularization. However, the only studies of delivered and non-perfused placentas found mean SWS values of 1.31 m/s [20], 1.23 m/s (60 Hz), 1.67 m/s (80 Hz), 1.74 m/s (90 Hz) and 1.80 m/s (120 Hz) [36]. These values are consistent with *in vivo* studies.

Folding the placenta in half could theoretically modify the elasticity of the latter, and SWAVE makes it possible to avoid this difficulty [36]. However, we did not observe any significant change in shear wave propagation at the interface between the two folded parts of the placenta.

Our study did not include a histopathological analysis of the placentas, as our main goal here was to determine the feasibility of this method as a first step. Moreover, routine anatomical analysis would not have made it possible to draw any further conclusions. In this type of study, it would be interesting to have more advanced analyses of the villous phenotypes, for example by morphometry, especially for comparison between normal and abnormal situations. The next step of this study is to compare the values of SWS, YM, and n in normal and abnormal placentas, first *ex vivo* and then *in vivo*.

Finally, our TE method using a vibrating plate is not suitable for clinical application to pregnant women. But this preliminary study enabled us to build a new device for *in vivo* examinations (S1 Appendix). With this new method the shear wave is followed in a different axis, and the question of folding the placenta no longer arises. For that reason, we have not continued these experiments on a larger sample of placentas. Our current experiments are applying our new device *in vivo*.

## Conclusion

In this *ex vivo* study, our frequency approach (measurement by TE) provided a new quantitative parameter, the “exponent n” of the fractional rheological model for assessing placental elasticity, in addition to the standard parameters such as YM or SWS at a particular frequency. This parameter n is variable for any given value of SWS and should be considered to provide additional information about the placental tissue. In the perspective of clinical application, it will be necessary to evaluate the performance of this frequency analysis, comparing the values of n in normal and abnormal situations.

## Supporting information

S1 Appendix New device for 2-D transient elastography. (A) This system has been developed for clinical application *in vivo*. (B) 2-D transient elastography system applied to a homogeneous elasticity phantom. (C) *Ex vivo* measurement on placenta.
(TIF)

S1 Table Clinical features and data from ten placentas. (XLSX)
