# Supplementary figures and images for: Measurement of shear wave speed dispersion in the placenta by transient elastography: A preliminary ex vivo study
Source: PLoS One. 2018 Apr 5;13(4):e0194309. doi: 10.1371/journal.pone.0194309 (PMC5886409; doi:10.1371/journal.pone.0194309)

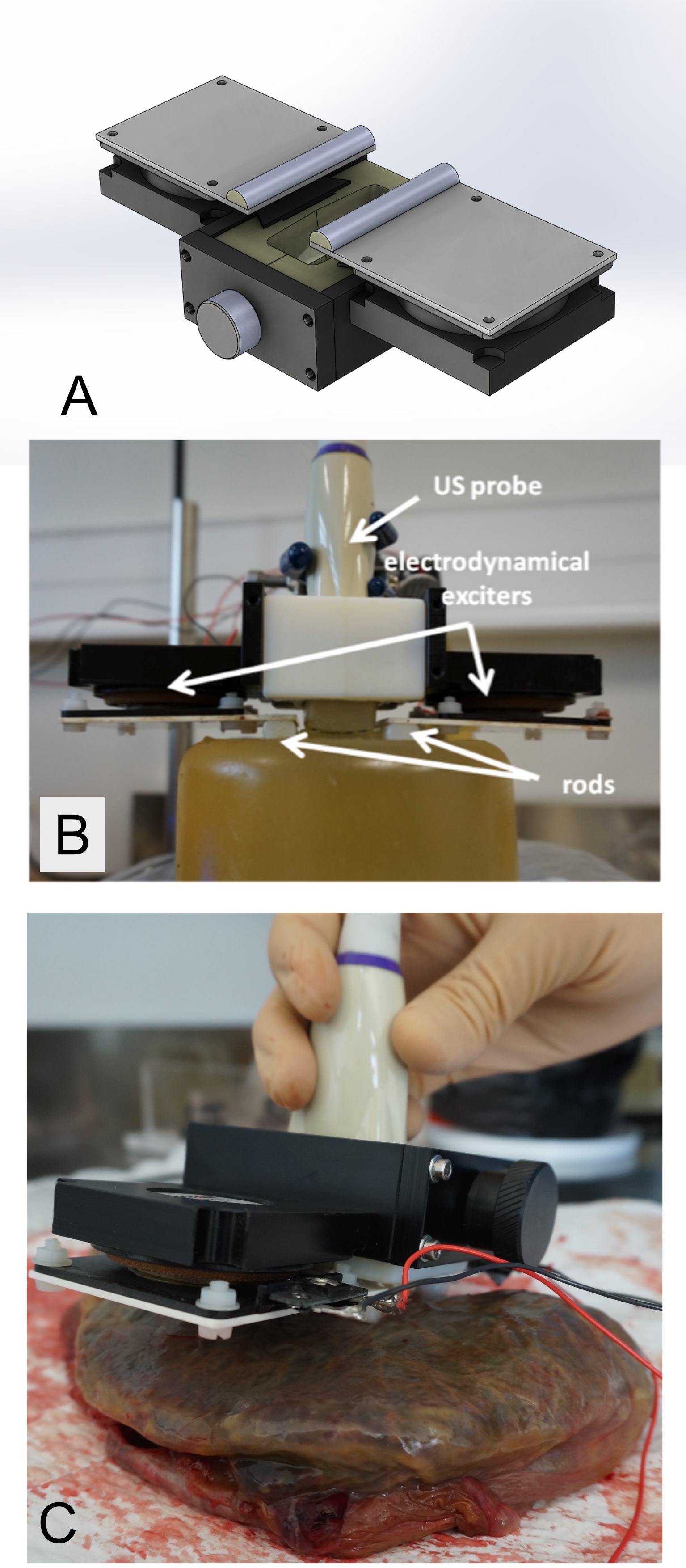

Supplement: S1 Appendix — (A) This system has been developed for clinical application in vivo. (B) 2-D transient elastography system applied to a homogeneous elasticity phantom. (C) Ex vivo measurement on placenta. (TIF) [file pone.0194309.s001.tif]
